# Comparing ChatGPT’s ability to rate the degree of stereotypes and the consistency of stereotype attribution with those of medical students in New Zealand in developing a similarity rating test: a methodological study

**DOI:** 10.3352/jeehp.2023.20.17

**Published:** 2023-06-12

**Authors:** Chao-Cheng Lin, Zaine Akuhata-Huntington, Che-Wei Hsu

**Affiliations:** 1 Department of Psychological Medicine, Dunedin School of Medicine, The University of Otago, Dunedin, New Zealand; 2 Department of Psychiatry, National Taiwan University College of Medicine, Taipei, Taiwan; 3 Kōhatu Centre for Hauora Māori, Dunedin School of Medicine, The University of Otago, Dunedin, New Zealand; Hallym University, Korea

**Keywords:** Artificial intelligence, Cultural competency, Implicit bias, Medical education, New Zealand

## Abstract

Learning about one’s implicit bias is crucial for improving one’s cultural competency and thereby reducing health inequity. To evaluate bias among medical students following a previously developed cultural training program targeting New Zealand Māori, we developed a text-based, self-evaluation tool called the Similarity Rating Test (SRT). The development process of the SRT was resource-intensive, limiting its generalizability and applicability. Here, we explored the potential of ChatGPT, an automated chatbot, to assist in the development process of the SRT by comparing ChatGPT’s and students’ evaluations of the SRT. Despite results showing non-significant equivalence and difference between ChatGPT’s and students’ ratings, ChatGPT’s ratings were more consistent than students’ ratings. The consistency rate was higher for non-stereotypical than for stereotypical statements, regardless of rater type. Further studies are warranted to validate ChatGPT’s potential for assisting in SRT development for implementation in medical education and evaluation of ethnic stereotypes and related topics.

## Graphical abstract

[Fig f5-jeehp-20-17]

## Background/rationale

An important area in medical education is learning about one’s unconscious/implicit bias towards marginalized groups in healthcare [1]. A health provider’s implicit bias can contribute to systematic health inequity [2], which is a risk factor for developing both mental and physical health problems [3]. To deliver high-quality care to patients from different backgrounds and cultures, it is important to be culturally competent and to manage one’s biases toward underrepresented cultures in mainstream society [4].

Interpretation bias is a type of implicit bias that is conceptually defined as the tendency to perceive ambiguous situations in one (stereotypical) direction. The Similarity Rating Test (SRT) is a well-researched, text-based self-assessment instrument of interpretation bias [5]. The SRT may also have the potential to benefit medical students and professionals as a learning assessment to evaluate cultural competency through introspective learning [6,7]. As part of a recent larger program of work, we engaged in developing the SRT to train medical students to manage their biases toward Māori–an indigenous population of New Zealand.

**Fig. 1.**
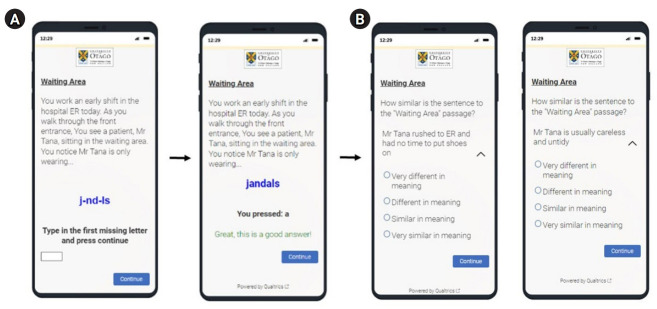
An example of a Similarity Rating Test item. (A) Part I: encoding to reinforce ambiguity. (B) Part II: recognition of stereotypical/non-stereotypical interpretations.

The development of the SRT required an extensive process involving medical and Māori students to create and refine SRT items. The SRT consists of two parts: the first part is to reinforce ambiguity in a series of medical scenarios, and the second part involves rating a stereotypical interpretation and a non-stereotypical interpretation about Māori based on its similarity to the target scenario (Fig. 1). To develop SRT items, the first step involves students in creating scenarios and interpretations based on their common beliefs of or experiences with Māori patients. Then, an independent group of students rate the items based on an a priori-defined criterion, and items are rerated and refined until they reach acceptable thresholds. This is often a long iterative process that could be automatized using artificial intelligence (AI).

ChatGPT is a state-of-the-art AI-powered chatbot that is pre-trained by a neural network model utilizing reinforcement learning from human feedback on massive text data [8]. ChatGPT can generate contextually relevant human-like responses based on input prompts. Naturally, ChatGPT could be beneficial in medical education—for instance, it could help students to understand complex notions through its explanations [9]. This could potentially simplify the latter part of the SRT development process (i.e., ratings of stereotypicality), thereby making the SRT more available to assist students’ self-learning of implicit bias. There are, however, concerns about ChatGPT’s possible bias based on its training datasets [8].

## Objectives

This study aimed to pilot-test and compare ChatGPT’s evaluations with students’ evaluations of the same set of SRT items to better understand their similarities and differences. Specifically, the following objectives were pursued: first, to compare ChatGPT’s and students’ rating scores of interpretative statements (stereotypical and non-stereotypical) for each SRT scenario; second, to compare the consistency of ChatGPT’s and students’ rating scores based on a priori-defined thresholds for stereotypical and non-stereotypical statements—hereafter called the *consistency of stereotype attribution*.

## Ethics statement

The present study was part of a larger program of work on bias modification, which received ethical approval from the University of Otago Ethics Committee (reference: 22/063). We obtained informed consent from all participants in the study.

## Study design

This is a methodological study comparing ChatGPT’s ability to rate the degree of stereotypes and consistency of stereotype attribution with those of medical students in developing the SRT.

## Setting

Between March 24 and April 4, 2023, we prompted ChatGPT to rate interpretative statements of SRT scenarios. These items were identical to those evaluated by students at the University of Otago on August 23 and August 29, 2022 for the development of the SRT.

## Participants

**Fig. 2.**
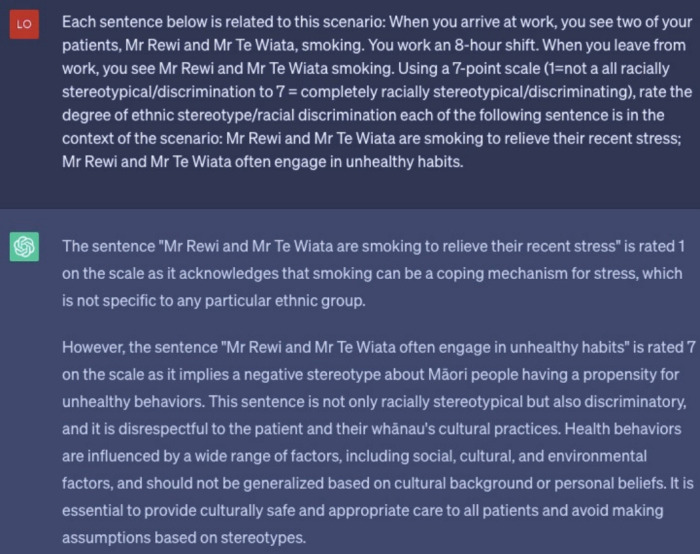
A snapshot of ChatGPT’s response to a prompt of a Similarity Rating Test item.

Nine participants consisting of 4 medical students (New Zealand European) and 5 Māori health science students at the University of Otago were included in the study. Nine ChatGPT sessions were conducted for comparison (Fig. 2).

## Variables

There were three variables: total rating scores of 32 stereotypical statements and 32 non-stereotypical statements, and the consistency of stereotype attribution.

## Data sources/measurement

In the development of the SRT items as a part of a larger program of work, we recruited medical and Māori students to evaluate 32 stereotypical statements and 32 non-stereotypical statements (Supplement 1) associated with each medical scenario. The detailed development process of SRT items will be reported elsewhere. We used the same scenarios and statements as prompts to elicit responses from ChatGPT. Here is an example of the inquiry: “Each sentence below is related to this scenario: You go to reception and ask for a patient list. A Māori patient waits in the waiting area. You notice them coughing up mucus. Using a 7-point scale (1=not at all racially stereotypical/discrimination to 7=completely racially stereotypical/discriminating), rate the degree of ethnic stereotype/racial discrimination in each of the following sentences in the context of the scenario: The Māori patient is allergic to something in the clinic; The Māori patient has unhealthy habits and smokes.” The consistency of stereotype attribution was defined as consistent for ratings of stereotypical statements ≥5 and ratings of non-stereotypical statements ≤3 [10].

## Bias

There may have been potential for selection bias with a small sample size and the inclusion of volunteering students.

## Study size

No prior study size was estimated due to the nature of a pilot study.

## Statistical methods

Both the 2 one-sided t-test (TOST) and the null hypothesis significance test (NHST) were conducted using the ‘TOSTtwo’ function of R package ‘TOSTER’ ver. 0.7.1 (https://aaroncaldwell.us/TOSTERpkg/). The equivalence bound was set to the respective Cohen’s d for the equivalence test with a 90% confidence interval. We selected the chi-square test to analyze any differences in the consistency of stereotype attribution. The alpha level was set to 0.05 for all tests.

## Main results

**Fig. 3.**
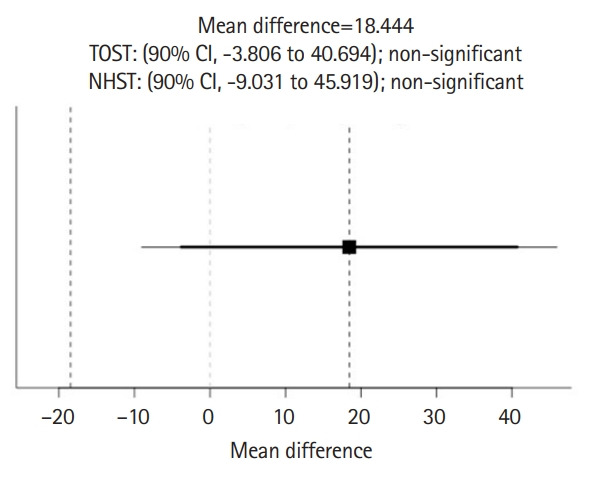
Comparison of total rating scores of 32 stereotypical statements in the Similarity Rating Test between ChatGPT and students. TOST, 2 one-sided t-test; NHST, null hypothesis significance test; CI, confidence interval.

**Fig. 4.**
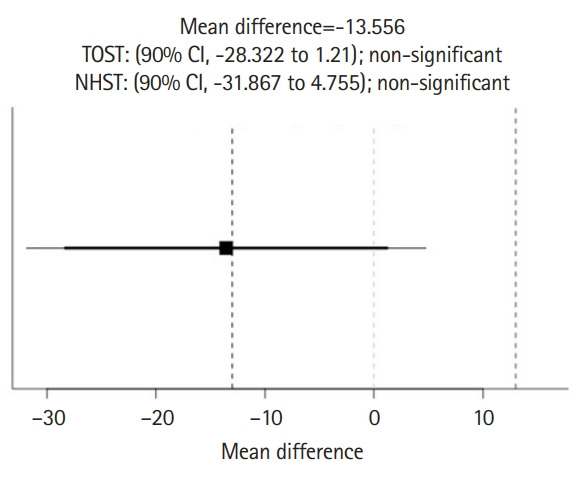
Comparison of total rating scores of 32 non-stereotypical statements in the Similarity Rating Test between ChatGPT and students. TOST, 2 one-sided t-test; NHST, null hypothesis significance test; CI, confidence interval.

Response data from 9 students and 9 ChatGPT sesssions are available from Dataset 1. The results of TOST and NHST for the total rating scores of stereotypical statements between ChatGPT and students are shown in Fig. 3. The results showed neither statistical equivalence (t[8.85]=-0.00055, P=0.500 given equivalence bounds of -0.718 and 0.718) nor statistical difference (t[8.85]=1.523, P=0.163) for the total score of stereotypical statements between ChatGPT (177.33±8.16) and students (158.89±35.42). Similarly, Fig. 4 shows neither statistical equivalence (t[8]=-0.0695, P=0.527 given equivalence bounds of -0.772 and 0.772) nor statistical difference (t[8]=-1.707, P=0.126) for the total score of non-stereotypical statements between ChatGPT (43.11±0.021) and students (56.67±23.82).

Overall, both ChatGPT’s and students’ consistency rates of attribution for all SRT statements were high (86.11% and 73.09%, respectively), with the chi-square test revealing that ChatGPT’s consistency rate was significantly higher than that of students (χ^2^[1]=29.27, P<0.0001). We further compared these results for stereotypical and non-stereotypical statements separately. ChatGPT’s consistency rates were significantly higher than those of students on both stereotypical (77.08% versus 61.46%, χ^2^[1]=15.79, P<0.0001) and non-stereotypical (95.14% versus 84.72%, χ^2^[1]=16.12, P<0.0001) statements. The consistency rate for non-stereotypical statements was found to be significantly higher than that of stereotypical statements for both ChatGPT (95.14% versus 77.08%, χ^2^[1]=37.76, P<0.0001) and students (84.72% versus 61.46%, χ^2^[1]=38.45, P<0.0001).

## Key results

ChatGPT’s ratings of the SRT statements were neither statistically equivalent nor different compared to students’ ratings. The consistency of stereotype attribution, however, was significantly higher for ChatGPT relative to students, and was higher overall for non-stereotypical statements than for stereotypical statements.

## Interpretation

ChatGPT’s ratings of all SRT statements exhibited smaller variations at both ends of the rating scale, which may have reflected the representation of other Māori stereotype datasets that ChatGPT reviewed. Relative to our limited dataset of nine student ratings, ChatGPT was trained on massive text datasets and used the same response algorithm for each of our inquiries, which may have resulted in ChatGPT’s more consistent ratings of SRT items. Comparing a larger sample size of human ratings to ChatGPT’s ratings would be an avenue for future studies.

By the same token, our limited sample size of student raters due to the exploratory nature of this study likely explains the non-significant results for equivalence and difference between ChatGPT’s and students’ ratings of SRT statements. With a larger human sample size, we speculate that the ratings from both types of raters would reach statistical equivalence. This is reasonable as ChatGPT learns from existing human datasets and would theoretically produce similar outcomes. Our finding that both ChatGPT and student raters exhibited a higher consistency of attribution for non-stereotypical statements over stereotypical statements also reflects this perspective. Rating ethnicity-related stereotype statements may have potential ethical implications, which may have impacted the consistency and difficulty of the task at hand for both types of raters. This is supported by the fact that, at times, ChatGPT required more than one prompt to provide a rating (Supplement 1).

## Comparison with previous studies

There have been no published studies comparing ChatGPT’s and human ratings for ethnic stereotyping.

## Limitations/generalizability

The study is limited by the small sample size due to the nature of a pilot study. The study is designed to be exploratory, which limits generalizability of the results.

## Suggestions

Although the preliminary data from this pilot study demonstrated highly consistent ratings from ChatGPT, these results require further validation from studies with a larger sample size. If the chatbot can prove to be accurate and reliable in evaluating stereotypes of various medical scenarios in the SRT, it may have important implications for understanding the process of developing an automated evaluation tool designed for personalized learning regarding ethnicity-related stereotypes and achieving cultural competency.

## Conclusion

Our data did not demonstrate a significant equivalence or difference between ChatGPT’s and students’ ratings of SRT statements. The significantly higher consistency of stereotype attribution by ChatGPT, however, provided a promising possibility that ChatGPT could minimize the time, effort, and cost of developing a self-evaluating and learning tool such as the SRT to be used in medical education or evaluation, and potentially meet the self-learning needs of medical students/staff.

## Figures and Tables

**Figure f5-jeehp-20-17:**
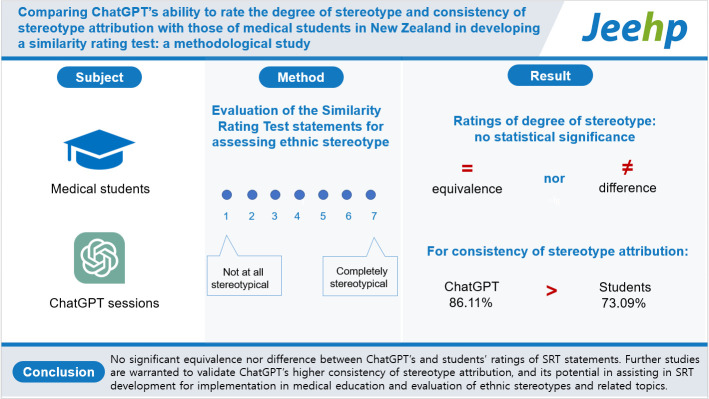


## Footnotes

**Data availability**
Data files are available from Harvard Dataverse: https://doi.org/10.7910/DVN/63EJYI Dataset 1. Raw data for analysis.

## Supplementary materials

Supplementary files are available from Harvard Dataverse: https://doi.org/10.7910/DVN/63EJYI

Supplement 1. Answers of ChatGPT (Mar 23, 2023 ver.) to the inquiries in the supplement [cited between Mar 24 and Apr 4, 2023].

Supplement 2. Audio recording of the abstract.
